# Immunolocalization of FGF-2 and VEGF in rat periodontal ligament during experimental tooth movement

**DOI:** 10.1590/2176-9451.19.3.067-074.oar

**Published:** 2014

**Authors:** Milene Freitas Lima Salomão, Sílvia Regina de Almeida Reis, Vera Lúcia Costa Vale, Cintia de Vasconcellos Machado, Roberto Meyer, Ivana Lucia Oliveira Nascimento

**Affiliations:** 1 Assistant professor, School of Medicine and Public Health of Bahia (EBMSP).; 2 Adjunct professor, EBMSP.; 3 Full professor, University of the State of Bahia (UNEB).; 4 Visiting professor, Brazilian Dental Association/Bahia (ABO-BA).; 5 Associate Professor, UFBA.

**Keywords:** Periodontal ligament, Orthodontics, Vascular endothelial growth factor A, Fibroblast growth factor 1

## Abstract

**Objective:**

This article aimed at identifying the expression of fibroblast growth factor-2 (FGF-2) and vascular endothelial growth factor (VEGF) in the tension and pressure areas of rat periodontal ligament, in different periods of experimental orthodontic tooth movement.

**Methods:**

An orthodontic force of 0.5 N was applied to the upper right first molar of 18 male Wistar rats for periods of 3 (group I), 7 (group II) and 14 days (group III). The counter-side first molar was used as a control. The animals were euthanized at the aforementioned time periods, and their maxillary bone was removed and fixed. After demineralization, the specimens were histologically processed and embedded in paraffin. FGF-2 and VEGF expressions were studied through immunohistochemistry and morphological analysis.

**Results:**

The experimental side showed a higher expression of both FGF-2 and VEGF in all groups, when compared with the control side (P < 0.05). Statistically significant differences were also found between the tension and pressure areas in the experimental side.

**Conclusion:**

Both FGF-2 and VEGF are expressed in rat periodontal tissue. Additionally, these growth factors are upregulated when orthodontic forces are applied, thereby suggesting that they play an important role in changes that occur in periodontal tissue during orthodontic movement.

## Abstract (pt)

**Objetivo:**

o objetivo desse estudo foi identificar a expressão do fator de crescimento de fibroblastos 2 (FGF-2) e do fator de crescimento vascular endotelial (VEGF) nos lados de tensão e pressão do ligamento periodontal de ratos, durante movimento ortodôntico experimental, em diferentes períodos de tempo.

**Métodos:**

uma força ortodôntica de 0,5N foi aplicada no primeiro molar superior direito de 18 ratos Wistar machos, por períodos de 3 (grupo I), 7 (grupo II) e 14 dias (grupo III). O primeiro molar do lado oposto foi utilizado como controle. Os animais foram sacrificados nos períodos de tempo mencionados, sendo a arcada superior removida e fixada. Após a desmineralização, os espécimes foram processados histologicamente e embebidos em parafina. A expressão do FGF-2 e do VEGF foram estudadas por meio de análise imuno-histoquímica.

**Resultados:**

o ligamento periodontal dos dentes submetidos à movimentação ortodôntica mostraram maior expressão tanto de FGF-2 quanto de VEGF, em todos os grupos experimentais, quando comparados com os dentes do lado controle (p < 0,05). Diferenças estatisticamente significativas entre os lados de tensão e pressão também foram encontradas nos dentes submetidos à movimentação ortodôntica.

**Conclusões:**

tanto o FGF-2 quanto o VEGF são expressos no tecido periodontal de ratos, e esses fatores de crescimento são aumentados quando forças ortodônticas são aplicadas, sugerindo que esses desempenham um papel importante na reorganização do periodonto durante o movimento ortodôntico.

## INTRODUCTION

Orthodontic tooth movement is achieved by remodeling the alveolar bone and periodontal ligament (PDL) in response to mechanical loading.^17^ It is a highly sophisticated biological process that leads to local inflammation, with vascular, cellular and extracellular matrix (ECM) alterations that allow remodeling events and, ultimately, tooth displacement.^17,37^ Orthodontic tooth movement is characterized by the abrupt creation of compression and tension sides in the PDL, with a repeated process of alveolar bone resorption on the pressure side and new bone formation on the tension side.^3,6,35^

Although the exact mechanism of periodontal tissue remodeling is not clearly understood, a milieu of cytokines, growth factors, neurotransmitters, ECM components, colony-stimulating factors and inflammatory mediators have been reported to be synthesized and released in PDL during orthodontic movement.^2,7,17,25,28,36^ These molecules interact with various dental and paradental cells and stimulate them to initiate and sustain tissue remodeling, inducing bone deposition and resorption.^6,42^

Continuous orthodontic forces can exert pressure that compromises the integrity of the vascular compartment in PDL. Over-compression results in ischemia, gradual reduction of capillaries, presence of thrombi, interruption of nutrition and cell death^21,29^with almost unavoidable formation of a necrotic or hyaline zone, mainly on the pressure side.^27,38^ In contrast, dilated blood vessels were found in the tension side.^33,38^

These vascular alterations can be mediated by different growth factors, such as fibroblast growth factor-2 (FGF-2) and vascular endothelial growth factor (VEGF). FGF-2, also known as basic FGF, is a potent angiogenic factor that shows increased expression in hypoxic conditions and during wound healing.^5,15^ This growth factor enhances endothelial cell proliferation and induces endothelial cell sprouting.^3^ Likewise, FGF-2 is a component of bone matrix and plays an important role in regulating bone remodeling.^13,19^

VEGF is considered the most important regulator of vasculogenesis and angiogenesis in physiological as well as in pathological conditions.^4,9^ *In vivo*, VEGF enhances vascular permeability and induces potent angiogenic responses.^8,10^ There is solid evidence for a functional link between vasculogenesis and bone development.^41^ Furthermore, VEGF may participate in the regulation of bone metabolism and wound healing during orthodontic tooth movement.^16,23^ This growth factor has the ability to induce functional osteoclasts when injected in PDL, thereby increasing the rate of tooth movement in mice.^16^

Thus, this study was designed to assess the expression levels of FGF-2 and VEGF in rat periodontal tissue submitted to mechanical forces in an experimental model of orthodontic tooth movement.

## MATERIAL AND METHODS

### Animal model and experimental orthodontic tooth movement

All experiments were conducted according to the guidelines of the Ethics Committee on Animal Use from the Federal University of Bahia (Brazil) where this study was submitted and approved.

**Figure 1:**
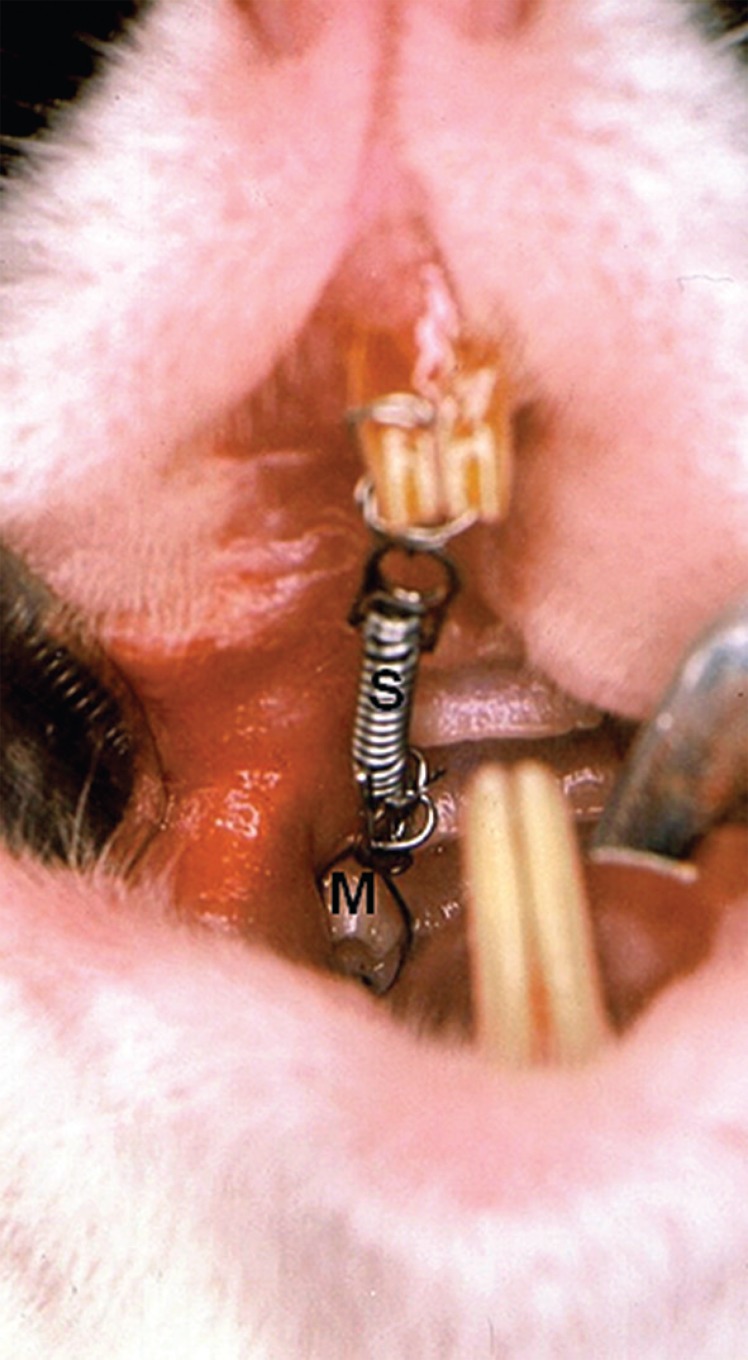
Occlusal view of orthodontic appliance placed on rat upper right first molar. The closed-coil spring (S) is attached to the molar (M) and incisor.

The study sample comprised 18 male Wistar rats aged between 60 ± 5 days (mean ± SD), with a mean weight of 170 g. The upper right first molar in each animal was mesially moved by means of a closed coil spring (3M Brasil, Sumaré, Brazil) which was fixed to the upper incisor from the same side (Fig 1), as previously described by Heller and Nanda.^11^ Grooves were made on the incisors to support the appliance. Forces of 0.5 N were applied for periods of 3 (group I, n = 6), 7 (group II, n = 6) and 14 days (group III, n = 6). The intensity of force was assessed using a dynamometer (Dentaurum Brasil, São Paulo, Brazil) while the spring was being fixed and then every day during the three different experimental periods. The upper left first molar, which was not subjected to any orthodontic movement, served as control. The orthodontic appliance was fixed and activated under anesthesia induced by intraperitoneal injection of ketamine (0.12 ml/100 g) and xylazine (0.06 ml/100 g). The animals had access to food and water *ad libitum*, and were kept on a reversed 12-h light/12-h dark cycle (dark period 08.00-20.00 h).

### Tissue processing

At the end of each experimental period, the animals were euthanized under deep anesthesia. The maxillary bone was removed, sagittally sectioned on the midline and fixed in 4% buffered paraformaldehyde for 24 h. The specimens were decalcified in 10% EDTA at room temperature (pH 7.2) for 12 weeks, processed histologically and paraffin embedded. Sections of 5 µm, parallel to the long axis of the first upper molar, were obtained and mounted on glass slides.

### Morphological and immunohistochemical analysis

For morphological analysis, one section of each sample was stained with hematoxylin and eosin and analyzed by light microscopy. For immunohistochemical assay, a streptavidin-biotin complex (LSAB, Dako Cytomation, Carpinteria, USA) was used. For detection of FGF-2 and VEGF, polyclonal anti-FGF-2 (dilution 1:1000; clone 147; Santa Cruz Biotechnology, Santa Cruz, CA) and monoclonal anti-VEGF (dilution 1:50; clone C-1; Santa Cruz Biotechnology, Santa Cruz, CA) antibodies were respectively used. The sections were dewaxed, rehydrated and washed in distilled water. The antigen retrieval was performed by enzymatic digestion with 1% trypsin (Sigma, Saint Louis, USA) for 20 min at 37°C. Endogenous peroxidase was blocked by treatment with 3% hydrogen peroxide for 10 min at 25°C. The slides were then incubated with the primary antibody in a humid chamber overnight at 4°C. Subsequently, the slides were washed with 1% PBS/BSA and incubated with biotinylated secondary antibodies (link reagent, Dako Cytomation, Carpinteria, USA) for 60 min at room temperature, followed by washing and incubation with the streptavidin-biotin-peroxidase complex. Diaminobenzidine (Dako Cytomation, Carpinteria, USA) was used as chromogen and the slides were counterstained with Harris hematoxylin (Sigma, Saint Louis, USA) for 15 seconds. Negative controls included replacement of primary antibodies with non-immune bovine serum albumin.

**Figure 2:**
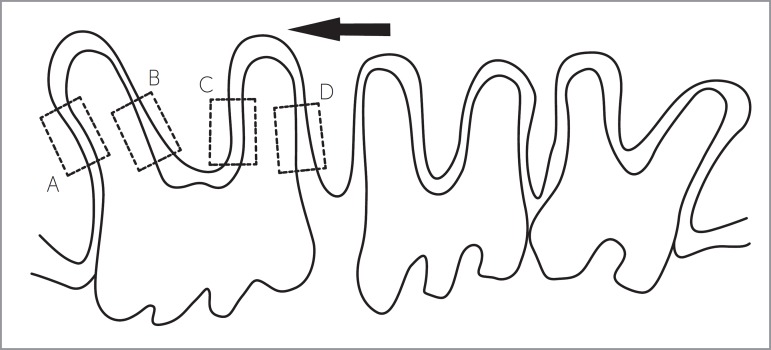
Diagrammatic representation of the areas chosen for morphological and immunohistochemical analyses. A and C correspond to pressure areas; B and D correspond to tension areas; arrow indicates direction of experimental orthodontic tooth movement.

Specific areas of the PDL were selected for morphological and immunohistochemical assessment. They corresponded to pressure and tension sides of the upper first molar submitted to orthodontic movement, as shown in Figure 2. The same areas in control teeth were chosen for analysis.

Quantitative analysis of immunohistochemistry was performed by means of a microscope (Axiolab, Zeiss, Germany) with a coupled camera (Axiocam HRP, Zeiss, Germany) linked to the Image J Software. Calibrations for each objective were performed using an image captured from calibration slides provided by the manufacturer. The slides were examined by random selection of two 0.1 mm^2^ areas. An experienced observer examined these images and identified DAB cells and excluded unstained tissue. Immunohistochemical staining was quantified by determining the percentage of the stained area. Thereafter, each area was captured under a final magnification of 400 x and saved in TIFF format.

### Statistical analysis

Wilcoxon Signed Ranks test was used to compare differences between the experimental and the control side as well as to compare the tension and the pressure areas within each experimental group. Kruskal-Wallis test and Dunn's post hoc test were used to compare the three experimental groups. Significance level was set at P < 0.05. Statistical analysis was performed with SPSS 17.0 for Windows.

## RESULTS

### Histology

**Figure 3:**
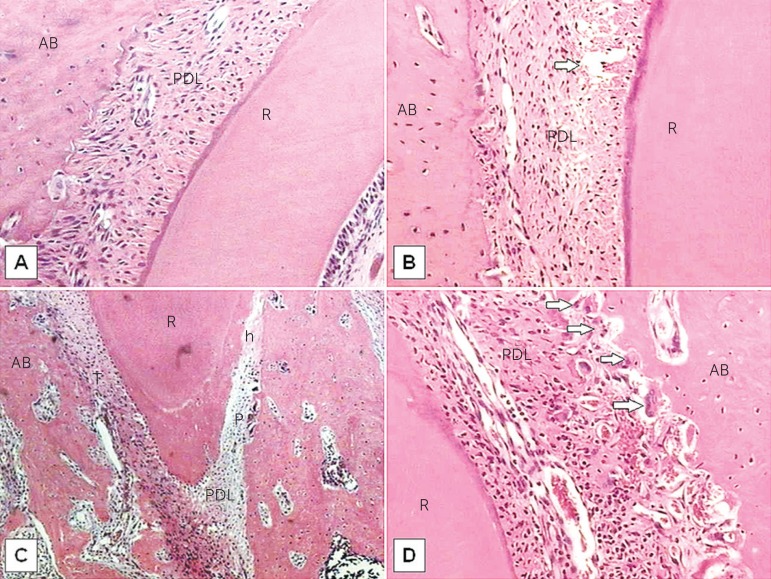
Histological findings in the control (A) and experimental groups (B-D) after 3 days of orthodontic tooth movement, stained with H&E. (A) PDL without signs of alteration (100x). (B) Disrupted fibers (arrow) observed on the tension side (100x). (C) Hyalinized areas (h) seen on the pressure side (40x). (D) Resorption lacunae with osteoclasts (arrows) observed on the pressure side (200x). AB indicates alveolar bone; PDL, periodontal ligament; R, root; T, tension side; P, pressure side.

All specimens comprising the control group exhibited a PDL without signs of alteration, as shown in Figure 3. In the groups submitted to orthodontic movement, marked alterations were observed on the PDL, especially in the interradicular space. Hyalinization areas were observed, mainly on the pressure side. Bone resorption was also observed with the presence of numerous osteoclasts. Most blood vessels collapsed and periodontal ligament fibers were rendered disorganized. On the tension side, the fibers were distended and sometimes disrupted. Hyperemic and dilated blood vessels were observed throughout the PDL extension on the tension side. Some areas of bone formation were found.

### Immunohistochemistry

**Figure 4:**
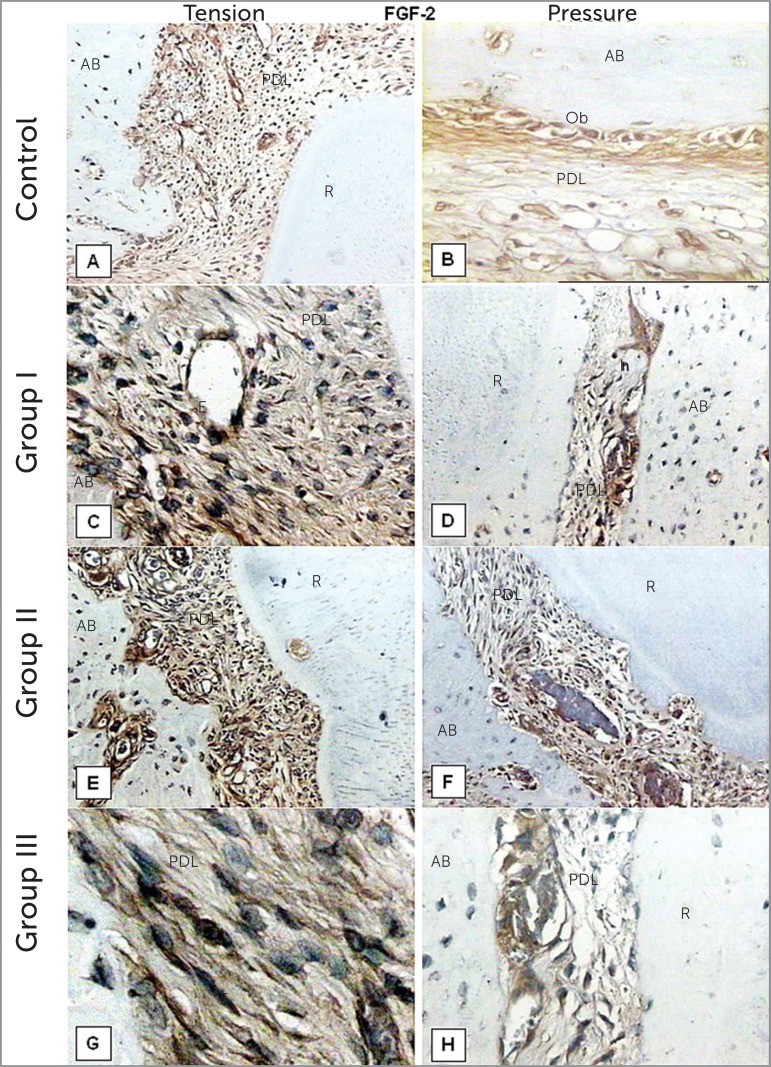
FGF-2 immunohistochemistry staining of the control (A,B) and experimental groups (C-H) after 3, 7 and 14 days of orthodontic tooth movement. (A) magnification of 40x; (B, C, H) 200x; (D, E, F) 100x; (G) 400x. AB indicates alveolar bone; PDL, periodontal ligament; R, root; Ob, osteoblasts; E, endothelial cells; h, hialinized area; F, fibroblasts.

**Figure 5:**
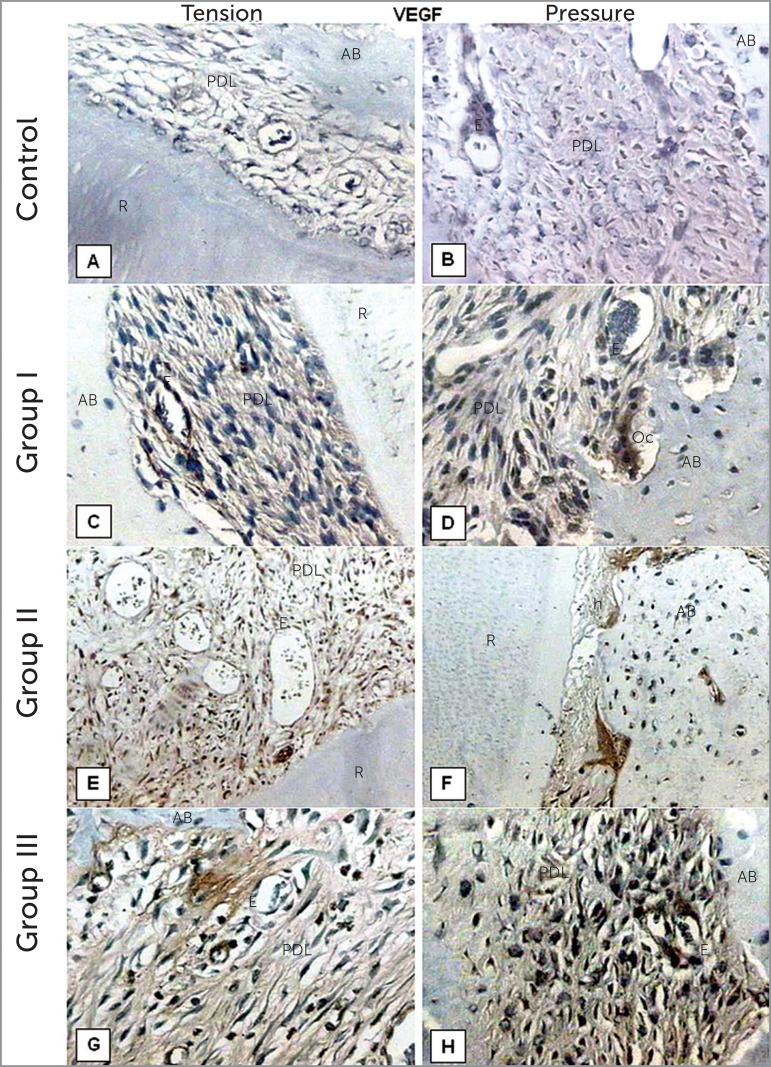
VEGF immunohistochemistry staining of the control (A,B) and experimental groups (C-H) after 3, 7 and 14 days of orthodontic tooth movement. (A, E, F) magnification of 100x; (B, C, D, G, H) 200x. AB indicates alveolar bone; PDL, periodontal ligament; R, root; E, endothelial cells; Oc, osteoclasts; h, hialinized area.

**Figure 6:**
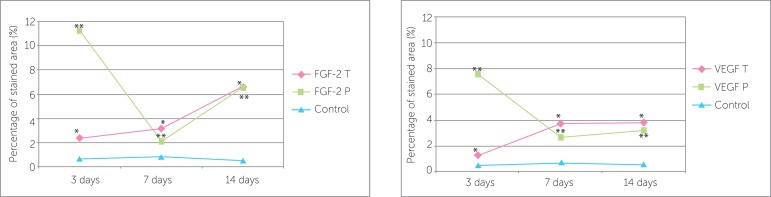
Percentage of FGF-2 and VEGF stained area (%) in the experimental and control groups, after 3, 7 and 14 days of orthodontic tooth movement; * and ** indicate statistically significant differences between experimental and control groups (P < 0.05). T indicates tension side; P, pressure side. FGF-2 control values: group I (0.68 ± 0.26); group II (0.80 ± 0.20); group III (0.50 ± 0.19). VEGF control values: group I (0.50 ± 0.21); group II (0.72 ± 0.10); group III (0.56 ± 0.22).

FGF-2 and VEGF immunoreactivity was detected in fibroblasts, osteoblasts, osteoclasts and endothelial cells in PDL of both the control and experimental sides (Figs 4 and 5). For FGF-2 expression, statistically significant differences were found between experimental and control groups at 3, 7 and 14 days (P < 0.05), as shown in Figure 6. When the pressure and tension sides were compared in the teeth that had undergone orthodontic movement, FGF-2 expression was significantly higher after 3 days of orthodontic movement on the pressure side, but not after 7 or 14 days (P < 0.05; Table 1). On the pressure side, all three experimental groups were statistically different for this growth factor, with group I showing the strongest expression (P < 0.05). On the tension side, FGF-2 expression was higher after 14 days of treatment, when compared with groups I and II (P < 0.05) (Table 1).

**Table 1:** Percentage of FGF-2 and VEGF stained areas (%) on tension and pressure sides of the three experimental groups.

	FGF-2		VEGF	
	Tension	Pression	Tension	Pressure
Group 1	2.40 ± 0.69^a^	11.25 ± 3.30^a*^	1.28 ± 0.52^a^	7.56 ± 2.34^a*^
Group 2	3.16 ± 0.96^a^	2.06 ± 0.94^b^	3.78 ± 1.04^b^	2.70 ± 0.54^b^
Group 3	6.63 ± 1.07^b^	6.56 ± 0.77^c^	3.83 ± 1.25^b^	3.20 ± 0.66^b^

Values account for mean and standard deviation (±). Different letters on the column indicate statistically significant difference between groups as well as between tension and pressure sides (P < 0.05).

There were also significant differences between the experimental and control groups after 3, 7 and 14 days of orthodontic movement for VEGF expression (P < 0.05; Fig 6). On the tension side, the expression of VEGF was statistically less in group I, when compared with groups II and III (P < 0.05). Conversely, on the pressure side, it was statistically higher on group I, when compared with the other groups (P < 0.05). When pressure and tension sides were compared, the expression of VEGF was higher on the pressure side after 3 days of orthodontic movement, but not after 7 and 14 days (P < 0.05) (Table 1).

## DISCUSSION

Orthodontic tooth movement is produced by mechanical forces that evoke biological responses. Mechanics and biology act together to produce desirable and predictable alterations in the form and function of the dentoalveolar complex.^18^ In this study, histological assessment revealed that experimental tooth movement induced periodontal remodeling. The main characteristic of the tension side was alveolar bone formation, whereas on the pressure side it was bone resorption, which is in accordance with other researches.^22^ Some hyalinized areas were observed, mainly on the pressure side. Previous studies show the presence of hyalinized areas in the periodontium after orthodontic tooth movement, even when light forces are used. Likewise, accumulation of osteoclasts near the hyalinization areas, which led to bone resorption on the pressure side, has been described.^39,40^ In our study, the presence of resorption lacunae containing osteoclasts on alveolar bone surfaces close to the hyalinized areas on the pressure side of PDL was observed. Cells such as macrophages, foreign body giant cells and osteoclasts remove this hyalinized necrotic tissue after a few days of force application, allowing tooth movement through the alveolar bone.^17^

We demonstrated that FGF-2 and VEGF are expressed in rat periodontal ligament cells, even at basal levels in control areas of PDL, thereby suggesting a constitutive production of these molecules by PDL cells. The expression of both FGF-2 and VEGF was assessed during experimental orthodontic tooth movement, indicating that upregulation of these cytokines could be associated with PDL remodeling. It is possible that this process suffers influence of these important angiogenic growth factors, since orthodontic forces alter blood flow in the periodontal region, initiating a cascade of biochemical and cellular processes that are responsible for these biological events.^12,23,24^

On the pressure side, PDL cells showed intense expression of FGF-2 three days after experimental tooth movement, which was concomitant with the observation of a higher number of osteoclasts and bone resorption in this group, thus indicating that this growth factor plays an important role during orthodontic movement. On day 7, a significant decrease of FGF-2 expression, as well as a lower number of osteoclasts were noted. One possible explanation is that there is dissipation of the applied orthodontic force due to tooth movement in the arch. A new increase in FGF-2 expression recorded on day 14 could be associated with PDL remodeling in this phase of orthodontic movement. FGF-2 has the ability to accelerate periodontal tissue regeneration at the final phase of tissue repair in alveolar bone defects by promoting angiogenesis and inducing growth of immature PDL cells.^24^

On the tension side, a gradual increase in FGF-2 was observed from day 3 to 14 of the induced orthodontic tooth movement, which is in agreement with the neoformation events observed in this region of PDL.^16,32^ After 14 days of orthodontic force application, a regeneration of periodontal tissue was observed, as well as a significant expression of FGF-2. It seems that FGF-2 is capable of inducing chemotaxis and mitogenesis of various PDL cells, thus, inducing tissue regeneration processes.^24,34^

There was a higher expression of VEGF on day 3 on the pressure side, probably due to the elevated number of osteoclasts observed in this area on the first days of experimental tooth movement. This could be explained by the ability of VEGF in inducing osteoclast differentiation.^1,14^ Continuous compressive forces enhance VEGF production and angiogenic activity in PDL cells, which may contribute to periodontal remodeling during orthodontic tooth movement.^23^ These reports suggest that VEGF expression in compressed periodontal tissue may play an important role in bone resorption, as well as in the promotion of angiogenesis in hyalinized tissues and adjacent areas on the pressure side. Moreover, through biological properties such as vascular permeability and chemotaxis, VEGF may provide the degenerated tissues with many cell types, for instance, fibroblasts, macrophages and multinucleated giant cells.^23^

On the tension side, there was a moderate expression of VEGF by the PDL cells, although an increase in this cytokine was observed along the three experimental periods. This is consistent with the demonstration of VEGF expression in osteoblasts on the tension side of mouse incisors and the predominance of alveolar bone formation that is characteristic of this region.^16,22^ Constitutive VEGF expression may contribute to PDL homeostasis by regulating blood circulation and bone metabolism.^23^

The higher expression of FGF-2 observed in this study during the first days of experimental tooth movement, when compared to VEGF, could be related to cellular events observed in the initial phase of inflammatory response resulting from the orthodontic force applied to the tooth. The generation of an acute inflammatory process, characteristic of orthodontic movement, may be responsible for the secretion of FGF-2.^20,26,30^ This growth factor is considered the most potent mitogen for periodontal cells and it may be important in wound healing, since it promotes angiogenesis and induces the development of immature PDL cells, thus, accelerating periodontal regeneration.^24,31^ Moreover, it seems that there is an optimal compressive force for VEGF production in PDL cells, and an excessive force results in decreased VEGF production.^23^

## CONCLUSION

The present study demonstrates that important alterations occur in PDL during experimental orthodontic tooth movement, in which bone formation and apposition on tension side and bone resorption on pressure side are the main events. The expression of both FGF-2 and VEGF is elevated during experimental orthodontic movement. It also varies with time, which can be related to the remodeling processes of PDL. FGF-2 levels were higher than VEGF levels in PDL during the first days of experimental orthodontic movement, thereby suggesting greater involvement of this protein in PDL remodeling. Moreover, the expression of these growth factors at basal levels in control areas of PDL suggests a constitutive production of these proteins by PDL cells.
